# Frailty and Increased Levels of Symptom Burden Can Predict the Presence of Each Other in HNSCC Patients

**DOI:** 10.3390/jcm13010212

**Published:** 2023-12-29

**Authors:** Viktor Kunz, Gunnar Wichmann, Theresa Wald, Andreas Dietz, Susanne Wiegand

**Affiliations:** Department of Otorhinolaryngology, Head and Neck Surgery, University Hospital Leipzig, 04103 Leipzig, Germany; gunnar.wichmann@medizin.uni-leipzig.de (G.W.); andreas.dietz@medizin.uni-leipzig.de (A.D.); susanne.wiegand@medizin.uni-leipzig.de (S.W.)

**Keywords:** frailty, geriatric questionnaire G8, Minimal Documentation System (MIDOS^2^), symptom burden, Edmonton Symptom Assessment System (ESAS), head and neck squamous cell carcinoma (HNSCC), head and neck cancer

## Abstract

Frailty is an important risk factor for adverse events (AEs), especially in elderly patients. Therefore, assessing frailty before therapy is recommended. In head and neck squamous cell carcinoma (HNSCC) patients, frailty is prognostic for severe postoperative complications and declining quality of life (QoL) after HNSCC treatment. Thus, assessment of frailty may help to identify individuals at risk for AE caused by oncologic therapy. We investigated the relationship between frailty and symptom burden to better understand their interaction and impact on HNSCC patients. In this prospectively designed cross-sectional study, the presence of frailty and symptom burden was assessed by using the Geriatric 8 (G8) and Minimal Documentation System (MIDOS^2^) questionnaires. A total of 59 consecutively accrued patients with a first diagnosis of HNSCC before therapy were evaluated. Patients were considered frail at a total G8 score ≤ 14. The MIDOS^2^ symptom burden score was considered pathological with a total score ≥ 4 or any severe symptom (=3). Statistical correlations were analyzed using Spearman and Pearson correlation. Receiver operator characteristic (ROC) curves were used to analyze the potential of predicting frailty and MIDOS^2^. *p*-values < 0.05 were considered significant. A total of 41 patients (69.5%) were considered frail, and 27 patients (45.8%) had increased symptom burden. “Tiredness” was the most common (overall rate 57.8%) and “Pain” was the most often stated “severe” symptom (5 patients, 8.5%). G8 and MIDOS^2^ correlated significantly (ρ = −0.487, *p* < 0.001; *r* = −0.423, *p* < 0.001). Frailty can be predicted by MIDOS^2^ symptom score (AUC = 0.808, 95% CI 0.698–0.917, *p* < 0.001). Vice versa, the G8 score can predict pathological symptom burden according to MIDOS^2^ (AUC = 0.750, 95% CI 0.622–0.878, *p* < 0.001). Conclusions: The strong link between frailty and increased symptom burden assessed by G8 or MIDOS^2^ indicates a coherence of both risk factors in HNSCC patients. Considering at least one of both scores might improve the identification of individuals at risk and achieve higher QoL and reduced complication rates by decision making for appropriate therapy regimens.

## 1. Introduction

The complex geriatric syndrome of frailty was identified as an important risk factor for various adverse effects (AE) in elderly patients, such as increased rates of postoperative complications [1,2] and increased rate of mortality [3] among patients with various types of malignancies. Patients older than 65 years represent the majority of cancer patients, making the group of elderly patients especially relevant and important for treatment in all oncologic disciplines [4]. Therefore, the assessment of frailty in elderly patients before either surgical [5] or oncologic [6] therapy is recommended. Elevated levels of symptom burden, on the other hand, mark an important risk factor for poor clinical outcomes [7] and the presence of psychological problems [8] in cancer patients as well.

In patients with head and neck squamous cell carcinoma (HNSCC), frailty was also identified as a prognostic marker for the occurrence of severe postoperative complications grade 3 or higher, according to the well-established Clavien–Dindo classification system [1], as well as a decline in self-reported quality of life (QoL) [9,10]. Thus, within this special vulnerable group of HNSCC patients, the assessment of frailty represents an important tool for identifying individuals at risk for both somatic and psycho-emotional adverse effects caused or modified in an unwanted way by oncologic treatment. 

Numerous diagnostic instruments and screening tools are available and validated for the assessment of frailty. In a recently published study by Gonzalez-Serrano et al., however, the comprehensive geriatric assessment (CGA) was shown to remain the gold standard and the best approach for assessment of frailty in patients with solid tumors [11]. The CGA, however, is known to be a time- and resource-consuming diagnostic instrument and is not broadly available for all patients that might benefit from a CGA, making these restrictions the main reasons for the high amount of alternative screening tools available. In this context, diagnostic screening tools for frailty, such as the Geriatric 8 questionnaire (G8) [12], were shown to provide good diagnostic accuracy in the detection of frailty, but failed to show benefits in clinical outcomes over the CGA for these patients [11].

To easily assess symptom burden in cancer patients, the revised version of the Minimal Documentation System (MIDOS^2^) is often used in Germany [13]. This tool represents the validated German version of the Edmonton Symptom Assessment System (ESAS) [14], which is validated for use in head and neck oncology itself [15], and measures symptoms on a self-reported basis. Recent studies were able to show the importance of symptom burden assessment, as increased levels of symptom burden in patients with recurrent or metastatic (R/M) HNSCC were found [16]. Symptom burden in HNSCC patients was also shown to be reduced depending on treatment response in patients with R/M HNSCC [17]. In this context, data for the simultaneous presence of symptom burden and frailty in patients with newly diagnosed HNSCC, prior to oncologic therapy, are scarce. 

As there is an overlap in items assessed in MIDOS^2^ and the G8, which also represents a well-established screening tool for frailty in HNSCC patients, our aim was to investigate the relationship between symptom burden according to MIDOS^2^ and frailty detected by G8 in elderly individuals with newly diagnosed HNSCC to further extend the knowledge of the impact these measures might have in HNSCC patients. Furthermore, the objective was to derive reliable identifiers before therapy that possibly contribute to a better understanding and distinction of HNSCC patients at risk for potential AE including severe adverse events (SAEs) caused by oncologic therapy. 

## 2. Patients and Methods

### 2.1. Statistical Considerations and Sample Size

This cross-sectional study was prospectively designed to assess the presence of frailty at baseline, as well as long-term follow-up, using the well-established G8 questionnaire and MIDOS^2^ for screening of frailty and symptom burden in patients with histologically proven and newly diagnosed HNSCC before therapy. The formula used for sample size calculation to identify frail HNSCC patients with pathologic symptom burden, according to MIDOS^2^, along with the error margin was

*n* = (*Z*_α_/2)^2^ × *P* × (1 − *P*)/*ε*^2^ (1)

*Z*_α_/2 = significance level α of the joint standard deviation for the 1 − α = 95% confidence interval (equaling the level of significance at 5%) = 1.96; *P* = prevalence of frailty expected (i.e., 40%, or 0.40); and *ε* = desired error margin (i.e., 18%, or 0.18). Therefore, *n* = (1.96 × 1.96 × 0.40 × (1 − 0.40))/0.18 × 0.18 = 28.5 or *n* = 29 cases at minimum. Considering 10% dropout due to incomplete questionnaires, a sample size of *n* = 32 patients per group would be required for the study, and *n* = 64 patients in total should be investigated to end up with a total sample of about *n* = 58 patients sufficient to answer the question about the predictability of complications according to the MIDOS^2^ when only the G8 questionnaire is used. 

### 2.2. Study Cohort

**Figure 1:**
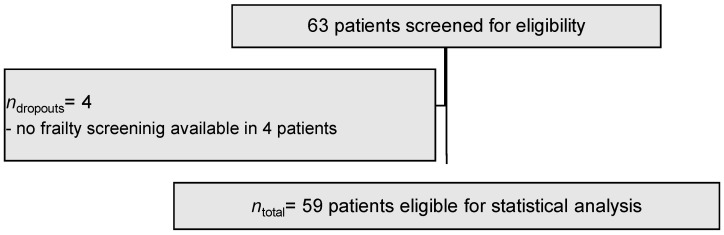
Patient selection.

The acquisition of data started in April 2022 and ended in January 2023. The G8 questionnaire and MIDOS^2^ were assessed in patients with first diagnosis of HNSCC who presented to the Department of Otorhinolaryngology, Head and Neck Surgery, University of Leipzig on a daily basis. The protocol was approved by the ethics committee of the Medical Faculty of Leipzig University (vote 125/22-ek). Study participants were consecutively accrued. The sample included patients of both sexes who were at the time of enrollment at least 18 years old and suspected of suffering from head and neck cancer. Besides being sufficiently fluent in the German language in written and spoken form, allowing for answering the items of the questionnaires, there were no further restrictions or selection criteria. Patients, who provided informed consent, received both questionnaires, G8 and MIDOS^2^, during the admission interview. Patient selection is shown in Figure 1: 

Out of 63 pre-screened patients, 4 patients had to be excluded because of lacking frailty screening prior to therapy, while 59 provided both completely filled out questionnaires. Therefore, the study cohort was composed of 59 consecutively accrued patients consequently including all cancer stages and a random sample of sexes and ages. All study participants had sufficient knowledge of the German language in spoken and written form. All patients completed both questionnaires themselves. As per study protocol, the enrollment of patients was terminated after recording the 63rd patient. 

### 2.3. Assessment of Frailty and Symptom Burden

We used the validated and well-established G8 questionnaire (see Supplementary Materials) to assess the presence of frailty in our cohort. The G8 questionnaire is a self-administered questionnaire consisting of 8 items. These items include questions about nutrition status, reduced mobility, neuropsychological problems, body mass index, intake of more than three daily drugs, self-estimated health status, and age. To prevent bias from leading questions or suggestions by an interviewer, the information is recorded by patients themselves. The maximum G8 score achievable equals 17 points. A total score ≤ 14 identifies patients who are suffering from frailty, whereas a total score of 15–17 points defines a robust individual. 

We used MIDOS^2^ (see Supplementary Materials) for the assessment of symptom burden in our cohort. This questionnaire assesses the patient’s symptoms using a symptom burden score (0 = no symptoms to 3 = strong symptoms). A total of ten symptom items (pain, nausea, vomitus, dyspnea, constipation, weakness, lack of appetite, tiredness, depression, anxiety), as well as a statement regarding general condition, are queried. Other symptoms can be added. The symptom burden score is then summed up to a total symptom score. A total score of ≥4 or any severe symptom score (=3) was defined as an increased level of symptom burden.

### 2.4. Statistical Analysis

All statistical analyses were performed using the software package for social sciences for MacOS (SPSS version 28.0.1; IBM Corp., Armonk, NY, USA). Statistical correlations were analyzed using Spearman and Pearson correlation. Prediction of frailty and MIDOS^2^ symptom burden score was performed with receiver operator characteristic (ROC) curves, using our chosen parameters. *p* values < 0.05 were deemed significant.

## 3. Results

### 3.1. Demographic Parameters and Study Cohort

The cohort eligible for statistical analysis consisted of 59 patients who provided complete data in both questionnaires. The cohort had a distribution of clinical and epidemiologic characteristics typical for predominantly male HNSCC patients as 83.1% of these patients were male. The mean age was 62.5 years (range 35 to 81). HNSCC of the oropharynx was the most frequent tumor site (23 patients, 39.0%) in our cohort. These and other descriptive parameters are summarized in Table 1.

### 3.2. Symptoms and Symptom Burden

**Figure 2:**
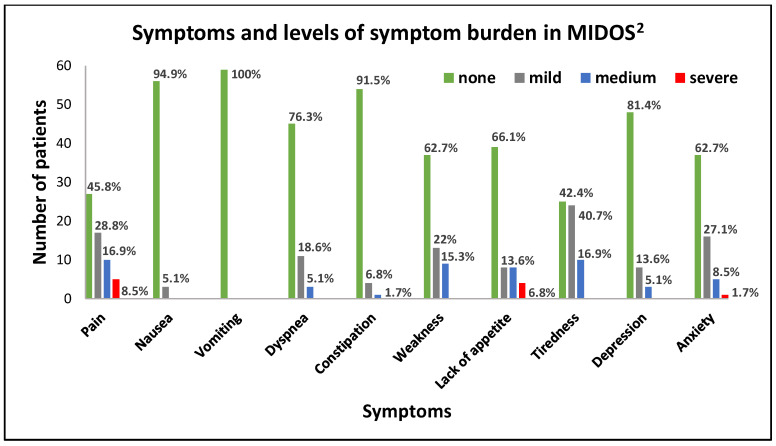
Descriptive statistics for symptoms and symptom burden in MIDOS^2^.

Tiredness was the most common symptom with an overall rate of 57.8%. Out of all symptom burden items classified as “severe”, “Pain” was most often reported in our cohort (5 patients, 8.5%), followed by “Lack of appetite” (4 patients, 6.8%). General condition was mainly stated as “good” (30 patients, 50.8%). A total of 21 patients (35.6%) reported their general condition as “medium”, 5 patients as “bad” (8.5%), and 2 patients as “very bad” (3.4%). Descriptive statistics for symptoms and symptom burden are shown in Figure 2.

### 3.3. Correlations and ROC Analysis

**Figure 3:**
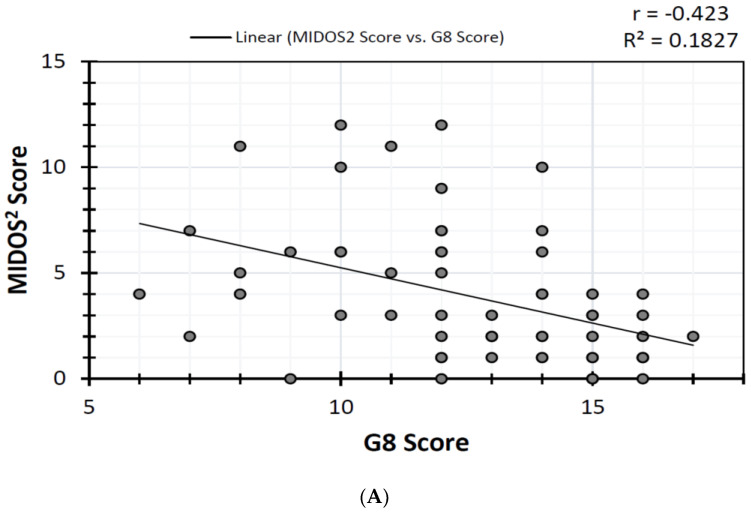

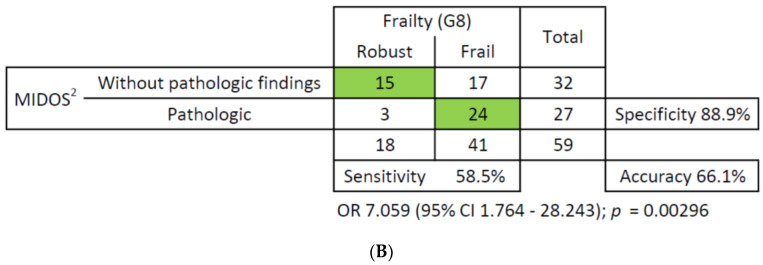
(**A**) Inverse correlation of MIDOS^2^ and G8 score. (**B**) Contingency table with specificity and sensitivity for increased symptom burden and frailty.

A significant negative, or inverse, correlation between the G8 questionnaire and MIDOS^2^ was found with Spearman’s (ρ = −0.487, *p* < 0.001) as well as Pearson’s (*r* = −0.423, *p* < 0.001). Correlations are shown in Figure 3A,B.

**Figure 4:**
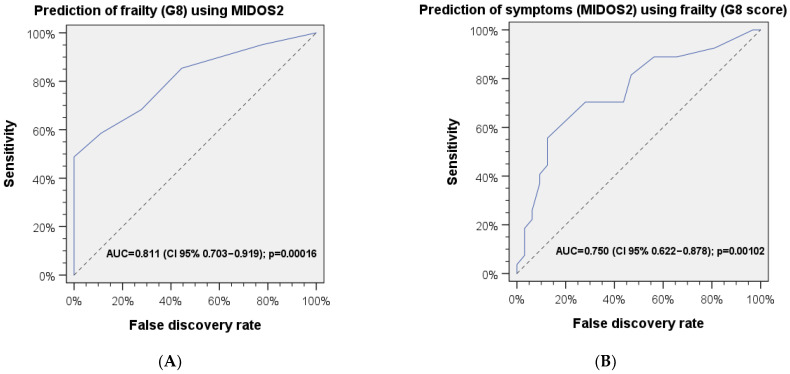
ROC analyses for prediction of (**A**) frailty with MIDOS^2^ symptom score and (**B**) MIDOS^2^ symptom score with frailty.

The presence of frailty, assessed with the G8 questionnaire, can be predicted significantly by MIDOS^2^ symptom score (AUC = 0.808, 95% CI 0.698–0.917, *p* < 0.001). ROC analysis for the prediction of frailty is shown in Figure 4. One local extremum for prediction of frailty was found at a MIDOS^2^ symptom score cut-off value = 1.5 (Youden’s *J* = 0.474, sensitivity = 85.4%, specificity = 55.6%). Another local extremum reflecting the optimal cut-off value was found at a MIDOS^2^ symptom score = 4 (Youden’s *J* = 0.488, sensitivity = 48.8%, specificity = 100%). Vice versa, the presence of increased symptom burden, assessed with the MIDOS^2^ symptom score, can be predicted significantly by frailty being present in the G8 score (AUC=.750, 95% CI 0.622–0.878, *p* = 0.001). The optimal cut-off value within the G8 questionnaire to predict a pathological MIDOS^2^ symptom score was at a G8 score = 13.75 (Youden’s *J* = 0.433, sensitivity = 81.5%, specificity = 53.1%). The results of ROC analyses are shown in Figure 4A,B.

## 4. Discussion

Confirming the findings of previous studies [18], “tiredness” and “pain” were also the most common or most often stated severe symptoms in our cohort. “Pain” was especially found to be the main symptom reported by patients one year after diagnosis and therapy of HNSCC [19]. In other cancer types, such as advanced pancreatic cancer, the mainly reported symptoms were “fatigue”, “nausea”, “anxiety”, and “shortness of breath” [20]. Our findings confirm the importance of both “tiredness” and “pain” within screening approaches for the most commonly shared symptoms and overall symptom burden in HNSCC patients. In this context, Mendoza et al. were able to show that poorly controlled symptom burden can have negative effects on treatment adherence and therapeutic outcomes in treatment-naive lung cancer patients [7]. On the other hand, Newcomb et al. were able to show that, in patients with solid and hematologic cancer, both psychological and physical symptoms are strongly present. At the same time, palliative care in order to address and target specific symptoms in these patients is rarely consulted [8]. Therefore, the assessment of symptom burden and resulting symptom control might also play an important role in HNSCC patients, which emphasizes the importance of assessing symptom burden and its proper management in these patients.

Our data indicate a strong correlation between the presence of frailty and increased levels of symptom burden, as both of these parameters show a significant inverse correlation in Spearman’s (ρ = −0.487, *p* < 0.001) and Pearson’s test (*r* = −0.423, *p* < 0.001). This strong negative correlation between MIDOS^2^ and G8 scores implies that the lower the G8 score, the higher the MIDOS^2^ score, and vice versa. Previous studies were also able to show a link between the presence of frailty and increased levels of symptom burden in patients with other severe medical conditions such as chronic kidney disease [21], liver cirrhosis [22], and R/M HNSCC [16]. Furthermore, our data show a strong coherence between the presence of frailty and MIDOS^2^ score in therapy-naive patients with newly diagnosed HNSCC, as, on the one hand, the presence of frailty can significantly be predicted by using the MIDOS^2^ score and, on the other hand, both of these risk factors also correlate significantly with each other (OR 7.059 [95% CI 1.764–28.243]; *p* = 0.00296). In this context, previous studies were able to show that frailty is an important risk factor for adverse effects after oncologic therapy, such as increased rates of severe postoperative complications—defined, for example, with a Clavien–Dindo classification score = 3 or more—after head and neck surgery [1,23], which emphasizes the importance of assessing the presence of frailty in HNSCC patients [5]. Assessment of frailty in cancer patients even seems to have an impact on clinical parameters. In patients with solid tumors, the assessment of frailty at baseline using a CGA was shown to be beneficial regarding their clinical outcomes [11]. On the other hand, assessment of frailty at baseline in elderly cancer patients provides multiple advantages such as enabling prognostication, risk stratification, and tailoring of treatment according to each individual’s needs and resources [4]. 

In summary, our results implicate that the presence of frailty, assessed with the G8 questionnaire, can also be predicted with increased levels of symptom burden, using the MIDOS^2^ symptom score, in HNSCC patients and vice versa. To our knowledge, there are no data available from any study or clinical trial in head and neck oncology that investigated this probably existing link or stated a coherence between frailty and increased levels of symptom burden in HNSCC patients, meaning that patients who suffer multiple symptoms or elevated levels of symptom burden might be significantly more likely to be frail at the same time. Interestingly, in this context, Amit et al. were able to show in HNSCC patients that symptom burden and QoL improve over time [24]. The question arises whether the same improvement can be observed in frail patients if frailty and increased levels of symptom burden are present simultaneously. Furthermore, as the presence of frailty in HNSCC patients is associated with poor outcomes after oncologic therapy [6], screening for increased symptom burden seems to be very important in these patients, and preventing potential complications by utilizing either G8 or MIDOS^2^ questionnaires seems to warrant the additional effort. Data regarding long-term changes in frailty after therapy in these patients, however, are scarce and should be investigated in future studies.

## 5. Limitations

Our study has some limitations that need to be discussed. First, our study was a single-center study and included some biases as the characteristics of the unselected consecutively accrued patients such as sex were heterogeneous, but, at the same time, provided an unselected “real life” dataset for our cohort of HNSCC patients. Secondly, this study was designed as a feasibility study excepting a wide error margin and therefore—despite adhering to the prospectively scheduled accrual—included a relatively low number of HNSCC patients. Third, the patients accepting the invitation to participate in the study and providing informed consent, and indeed delivering completely filled questionnaires were more likely to be highly motivated to do so and hence might not be that representative of the heterogeneous group of HNSCC patients as desired. Cancer staging was also not taken into consideration, which might result in higher levels of symptom burden and frailty in general, if one assumes that higher cancer stages correlate with higher levels of symptom burden. To our knowledge, there are no data available on HNSCC patients that prove or refute this assumption.

## 6. Conclusions

Our data show a significant correlation between frailty and increased levels of symptom burden. The strong link between frailty and increased levels of symptom burden in our data indicates coherence in HNSCC patients, as both of these risk factors can predict the presence of each other. A more profound understanding of the coherence between both of these risk factors might lead to better identification of individuals at risk by using at least one of both questionnaires, possibly enabling achievement of a better quality of life by reducing complication rates after oncologic therapy in the especially vulnerable group of HNSCC patients.

## Figures and Tables

**Table 1:** Descriptive parameters for sex, tumor site, age, symptom burden, and frailty.

		*n*	%
Sex	Male	49	83.1
	Female	10	16.9
HNSCC site	Oropharynx	23	39.0
	Larynx	15	25.4
	Oral cavity	13	22.0
	Hypopharynx	6	10.2
	CUP	2	3.4
Frailty (G8)	Robust	18	30.5
	Frail	41	69.5
Increased symptom burden	Yes	27	45.8
(MIDOS^2^)	No	32	54.2
	Age	MIDOS^2^ Score	G8
Mean	62.53	3.86	12.47
SD	10.23	3.27	2.70
Minimum	35	0	6
Maximum	81	12	17

## Data Availability

The datasets used and/or analyzed during the current study are available from the corresponding author upon reasonable request.

## Supplementary Materials

The following supporting information can be downloaded at: https://www.mdpi.com/article/10.3390/jcm13010212/s1.

## Abbreviations

*AUC*	Area under the curve
*CGA*	Comprehensive geriatric assessment
*CI*	Confidence interval
*CUP*	Cancer of unknown primary
*G8*	Geriatric 8 (screening instrument for the assessment of frailty)
*HNSCC*	Head and neck squamous cell carcinoma
*IBM*	International Business Machines Corporation
*J*	Youden’s J
*MIDOS^2^*	Minimal Documentation System 2
*n*	Sample size
*p*	*p*-value
ρ	Spearman’s rho
r	Pearson’s r
*ROC*	Receiver operating characteristic (used to assess the accuracy of tests in predictive models)
*SD*	Standard deviation
*SPSS*	Statistical Package for the Social Sciences
*Z_alpha_*	Standardized distance from mean (according to standard deviation units)
