# Supplementary material for: Frailty and Increased Levels of Symptom Burden Can Predict the Presence of Each Other in HNSCC Patients
Source: J Clin Med. 2023 Dec 29;13(1):212. doi: 10.3390/jcm13010212 (PMC10779894; doi:10.3390/jcm13010212)
Supplement: Supplementary file 1 [file jcm-13-00212-s001.zip › jcm-2778090-supplementary.pdf]

G8 questionnaire

	Items	Possible answers (score)
**A**	Has food intake declined over the past 3 months due to loss of appetite, digestive problems, chewing or swallowing difficulties?	0 : severe decrease in food intake
		1 : moderate decrease in food intake
		2 : no decrease in food intake
**B**	Weight loss during the last 3 months	0 : weight loss > 3 kg
		1 : does not know
		2 : weight loss between 1 and 3 kgs
		3 : no weight loss
**C**	Mobility	0 : bed or chair bound
		1 : able to get out of bed/chair but does not go out
		2 : goes out
**E**	Neuropsychological problems	0 : severe dementia or depression
		1 : mild dementia or depression
		2 : no psychological problems
**F**	Body Mass Index (BMI (weight in kg) / (height in m²)	0 : BMI < 19
		1 : BMI = 19 to BMI < 21
		2 : BMI = 21 to BMI < 23
		3 : BMI = 23 and > 23
**H**	Takes more than 3 medications per day	0 : yes
		1 : no
**P**	In comparison with other people of the same age, how does the patient consider his/her health status?	0 : not as good
		0.5 : does not know
		1 : as good
		2 : better
	Age	0 : >85
		1 : 80-85
		2 : <80
	**TOTAL SCORE**	**0 – 17**

Dear patient, you know your situation best yourself. Please fill out the questionnaire carefully. Please tick mark statements that apply most closely to your situation. You are welcome to ask your relatives or caregivers to help you. Thank you for your cooperation!	**Name** _ _ _ _ _ _ _ _ _ _ _ _ _ _ _<br>**Surname** _ _ _ _ _ _ _ _ _ _ _ _ _ _ _<br>**Date of birth** _ _/ _ _/ _ _ _ _<br>**Date** _ _/ _ _/ 20 _ _

Please mark the level of your symptoms today!				
**Pain**	0☐ none	1☐ mild	2☐ medium	3☐ severe Pain
**Nausea**	0☐ none	1☐ mild	2☐ medium	3☐ severe Nausea
**Vomiting**	0☐ none	1☐ mild	2☐ medium	3☐ severe Vomiting
**Dyspnea**	0☐ none	1☐ mild	2☐ medium	3☐ severe Dyspnea
**Constipation**	0☐ none	1☐ mild	2☐ medium	3☐ severe Constipation
**Weakness**	0☐ none	1☐ mild	2☐ medium	3☐ severe Weakness
**Lack of appetite**	0☐ none	1☐ mild	2☐ medium	3☐ severe Lack of appetite
**Tiredness**	0☐ none	1☐ mild	2☐ medium	3☐ severe Tiredness
**Depression**	0☐ none	1☐ mild	2☐ medium	3☐ severe Depression
**Fear**	0☐ none	1☐ mild	2☐ medium	3☐ severe Fear
**Other** ……………	0☐ none	1☐ mild	2☐ medium	3☐ severe …
**Other** …………..	0☐ none	1☐ mild	2☐ medium	3☐ severe …

Please mark how you feel today!					
**General condition**	☐ very bad	☐ bad	☐ medium	☐ good	☐ very good

Comments ……
……

**MIDOS²-Score** *(only to be filled out by medical professionals)*	∑
Screening performed on **… / … / 20 …** **Signature medical professional** ….………………….	
